# Androgen receptor (*AR*)-CAG trinucleotide repeat length and idiopathic male infertility: a case-control trial and a meta-analysis

**DOI:** 10.17179/excli2018-1744

**Published:** 2018-12-17

**Authors:** Narges Mobasseri, Faezeh Babaei, Mohammad Karimian, Hossein Nikzad

**Affiliations:** 1 Gametogenesis Research Center, Kashan University of Medical Sciences, Kashan, Iran

**Keywords:** male infertility, androgen receptor, CAG repeat, genetic association

## Abstract

CAG trinucleotide repeats in androgen receptor (*AR*) gene encode a polyglutamine tract in AR N-terminal transactivation domain. Studies have been conducted to evaluate the effect of CAG repeat length on male infertility, which have yielded contradictory results. This study aimed to explore the number of *AR*-CAG repeats in 150 fertile controls and 150 idiopathic infertile men, divided into four azoospermia, oligozoospermia, asthenozoospermia, and teratozoospermia subgroups. In addition, a meta-analysis was conducted based on previous studies to assess the association of the mentioned variation with male infertility in recent years. Polymerase chain reaction (PCR) targeting followed by an electrophoresis on polyacrylamide gel was used for *AR*-CAG genotype detecting. Moreover, a systematic search was performed in PubMed, Web of Science, Science Direct, and Google Scholar databases to collect eligible studies for meta-analysis purpose. According to the results, a significant association was observed between increased length of *AR*-CAG polymorphism and male infertility (*p*< 0.0001). Furthermore, there were similar significant associations in the azoospermia (*p*= 0.048), asthenozoospermia (*p*= 0.013) and teratozoospermia (*p*= 0.002) subgroups. In addition, meta-analysis on forty studies showed a significant association between *AR*-CAG polymorphism in the overall analysis (SMD= 0.199, 95 % CI= 0.112-0.287, *p*<0.001) and the Caucasian subgroup (SMD= 0.151, 95 % CI= 0.040-0.263, *p=* 0.008). Our results elucidated that long stretches of CAG repeat might lead to AR dysfunction, contributing to male infertility especially in the Caucasian population.

## Introduction

Infertility is a major health problem with associations to both genetic and environmental factors, affecting one-sixth of couples worldwide (Batiha et al., 2018[5]; Ge et al., 2014[19]). Approximately 50 % of infertility cases are attributed to male factors (Zorrilla and Yatsenko, 2013[67]). It is widely believed that several genetic factors may result in spermatogenesis failure and sperm impairment, including single gene mutations and chromosomal abnormalities (Li et al., 2014[31]). Androgens are essential hormones for male sex differentiation, normal development, spermatogenesis, and sexual behavior (Nilsson et al., 2015[43]). Similar to testosterone or dihydrotestosterone, androgens are generally secreted in male testes by Leydig cells and their activity are mediated by one of the steroid hormone receptors superfamily named androgen receptor (AR). This is the main starting point for regulating growth, differentiation, maturation of secondary sexual phenotypes, and natural spermatogenesis. Therefore, one cause of irregular and insufficient spermatogenesis, which can lead to male infertility, is the downregulation of endogenous androgen levels (Giagulli et al., 2014[20]). AR has a large communication and releases androgen signals among several body tissues, including prostate cells, seminal vesicle, melanocytes, keratinocytes, adipocytes, myocytes, hepatocytes, neuronal cells, and especially, obstructive and resistant Sertoli cells (O'Hara and Smith, 2015[45]). Therefore, androgen receptor gene mutations can lead to several deleterious disorders, including male infertility. Generally, the *AR* gene contains eight exons and seven introns, locating on chromosome Xq11-12 (Pan et al., 2016[46]). AR and other members of steroid receptor superfamily have three important basic domains, including a highly conserved central DNA-binding domain (DBD), a hinge region (HR), C-terminal ligand binding domain (LBD), and finally the N-terminal transactivation domain (NTD). The NTD, approximately 60 % of the AR protein length, contains a stretch of a variable number of CAG triads in the exon 1, which encodes a poly-glutamine (polyQ) tract (Khan et al., 2018[28]). The usual variation length of these repeats expands from 8 to 37 trinucleotides with a mean of 21 residues in the Caucasian population. However, there are significant ethnic variations in the allelic distribution of the *AR*-CAG polymorphism (Grigorova et al., 2017[21]; Casella et al., 2001[8]). Numerous studies have been performed to determine the association between the length of CAG repeats in the *AR* gene and male infertility. Nevertheless, the results have been inconsistent and challenging (Pastuszak et al., 2012[48]; Andersen et al., 2011[2]). In the present study, we investigated the distribution of the androgen receptor CAG polymorphism in the infertile population from Beheshti infertility center (Kashan, Iran), compared to normal samples from the same area. Moreover, we performed a meta-analysis in this field by including 40 eligible studies.

## Materials and Methods

### Subjects

In this case-control study, the blood samples of 150 idiopathic infertile men and 150 age-matched healthy fertile control men were prepared. All infertile samples were enrolled from the reproduction clinic of Beheshti hospital affiliated with Kashan University of Medical Sciences (Kashan, Iran). An accurate medical and reproductive history was taken from the infertile subjects through an interview, and all participants were examined for diseases such as orchitis, varicocele, maldescensus testis, immune, obstruction of vas deferens, infectious abnormalities, diabetes mellitus, drug abuse, abnormal hormones profile (LH, FSH, and testosterone), abnormal karyotype, and Y chromosome microdeletions. Furthermore, the participants were screened for genetic and familial diseases. Therefore, subjects with any of the above-mentioned disorders were excluded from the study. The subjects in the control group were also randomly selected from fertile men without any history of infertility, who had a minimum one child and completely normal sperm parameters. Finally, 2 ml peripheral blood samples were collected in impregnated EDTA_k+_ tubes from all participants and preserved at -20 ºC. An informed written consent was obtained from all the participants prior to the research and this study was approved by the Medical Research Ethics Committee of the Kashan University of Medical Sciences (Reference no. IR.KAUMS.REC.1396.24).

### Genomic DNA extraction and AR-CAG genotyping

**Figure 1:**
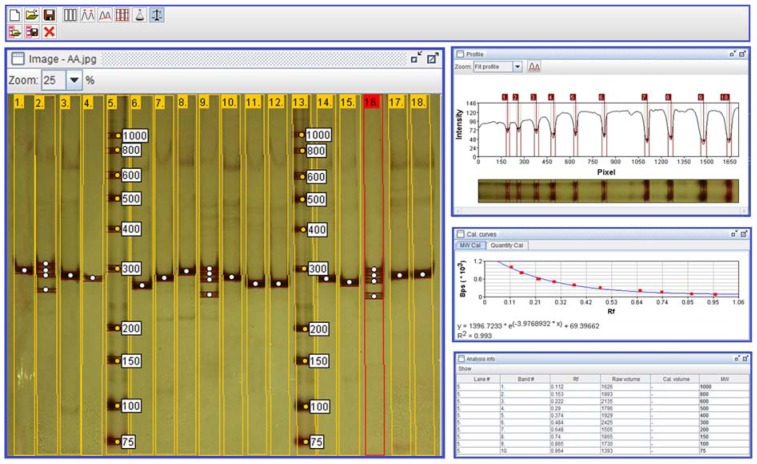
Genotyping of *AR*-CAG polymorphism by Gel Analyzer software. The genotype of the samples is determined on the polyacrylamide gel in the software, and the size of the bands is estimated by the ladder calibration.

Genomic DNA was extracted from the blood samples by DynaBio^TM^ genomic blood DNA extraction kit (Takapouzist Co., Tehran, Iran) and stored at -20 °C for future applications. Our researchers used the PCR technique for genotyping of the *AR*-CAG polymorphism. Sense (5'-CCAGAATCTGTTCCAGAGCGTG-3') and antisense (5'- GCTGTGAAGGTTGCTGTTCCTC-3') primers were designed surrounding the polymorphic region by Oligo7 software. In this study, the PCR was performed in a 20 µl total volume containing 10 µl 2X Taq PreMix, 0.35 µM of each primer, and 40 ng DNA template. A thermocycling protocol was carried out in peqSTAR thermal cycler (PeqLab, Erlangen, Germany) with the following program: the initial denaturation at 94 °C for five minutes, which continued by 40 repetitive cycles including the denaturation step at 94 °C for 45 seconds, annealing step at 57.7 °C for 45 seconds, extension step at 72 °C for 45 seconds, and final extension at 72 °C for five minutes. All PCR reagents were purchased from CinnaGen Company (Tehran, Iran). For rapid and accurate assessment of CAG repeat numbers, the DNA fragments were run on 8 % polyacrylamide gel and were visualized by AgNO_3 _staining protocol. After taking photos of the gels, Gel Analyzer 2010a software was used for *AR*-CAG repeat analyzing (Figure 1(Fig. 1)). To confirm the repeat lengths, some samples with different fragment sizes were sequenced by the forward primer in Bioneer Company (Daejeon, South Korea).

### Meta-analysis

Published reports were collected by a systematic search of electronic databases (PubMed, Google Scholar, ScienceDirect and Web of Science) up to August 2018, using the keywords of "male infertility", "androgen receptor", "CAG repeat", and "polymorphism". The selection criteria for publications were as follows: all reports must be on the association between CAG repeat length and male infertility in a case-control design; the case group should be included idiopathic infertile males with insufficient sperm parameters according to the criteria by the World Health Organization (WHO) in 1999[64] or earlier; abstracts and unpublished studies were omitted; and duplicate studies were considered only once. In addition, cited references from related reports, review articles, and other meta-analyses were screened as appropriate. The following information was extracted from each eligible study:

First author's name; publication year; country of origin; ethnicity; the number of samples, the mean and standard deviation (SD) of CAG repeat length for cases and controls, and association result. The ethnicity of publications was categorized into the two Asian and Caucasian groups (Table 1(Tab. 1); References in Table 1: Asatiani et al., 2003[3]; Badran et al., 2009[4]; Canale et al., 2005[7]; Casella et al., 2003[9]; Cram et al., 2000[11]; Dadze et al., 2000[12]; Dakouane-Giudicelli et al., 2006[13]; Dhillon and Husain, 2003[15]; Erasmuson et al., 2003[17]; Ferlin et al., 2004[18]; Hadjkacem et al., 2004[22]; Han et al., 2013[23]; Hiort et al., 2000[24]; Katagiri et al., 2006[27]; Lavery et al., 2005[30]; Lund et al., 2003[32]; Madgar et al., 2002[33]; Martínez-Garza et al., 2008[34]; Mengual et al., 2003[35]; Mifsud et al., 2001[37]; Mifsud et al., 2001[36]; Milatiner et al., 2004[38]; Mosaad et al., 2012[39]; Mostafa et al., 2012[40]; Pan et al., 2002[47]; Patrizio et al., 2001[49]; Peterlin et al., 2007[50]; Rajpert-De Meyts et al., 2002[52]; Ruhayel et al., 2004[53]; Saare et al., 2008[54]; Sasagawa et al., 2001[55]; Thangaraj et al., 2002[58]; Tse et al., 2003[59]; Tufan et al., 2005; Van Golde et al., 2002[61]; von Eckardstein et al., 2001[62]; Wallerand et al., 2001[63]; Zare-Karizi et al., 2016[66]). Data extraction was performed independently by two co-authors (NM and FB) until census was achieved for all data.

### Statistical analysis

In this case-control study, the mean value of various CAG repeats of infertile patients was compared to those infertile controls using the two-sample independent t-test. For subgroup analysis, one-way ANOVA test was used as appropriate. In addition, a *p*-value of less than 0.05 was considered statistically significant. The main goal of the meta-analysis was to determine the association between CAG repeat length and male infertility. Differences in repeat lengths between cases and controls were estimated by the overall standardized mean difference (SMD) and 95 % confidence interval (CI). Moreover, Cochran's Q statistic and I^2^ index were used to evaluate the heterogeneity. In this regard, *P*_heterogenity_ less than 0.1 was considered statistically significant. Furthermore, a random-effect model was employed when a true heterogeneity was observed; otherwise, a random-effect model was used. In addition, possible publication bias was analyzed by Begg's funnel plot and Egger test. Statistical analyses for case-control trial and meta-analysis were performed in SPSS version 20 and MedCalc statistical software, respectively.

## Results

### AR-CAG distribution

**Figure 2:**
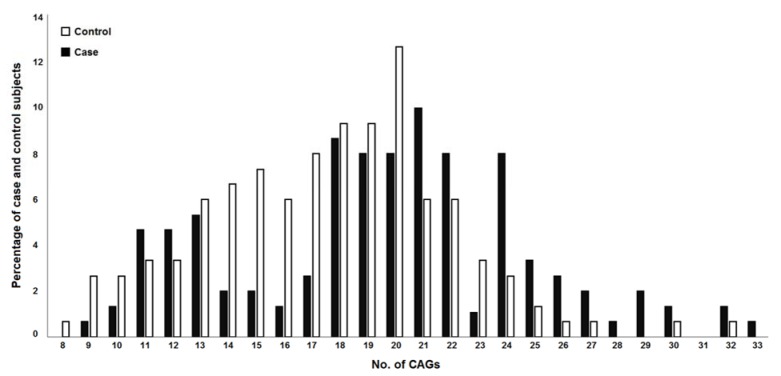
Frequencies of CAG allele in the fertile and infertile groups. The most frequent allele in fertile and infertile groups are 20 and 21 repeats, respectively.

**Figure 3:**
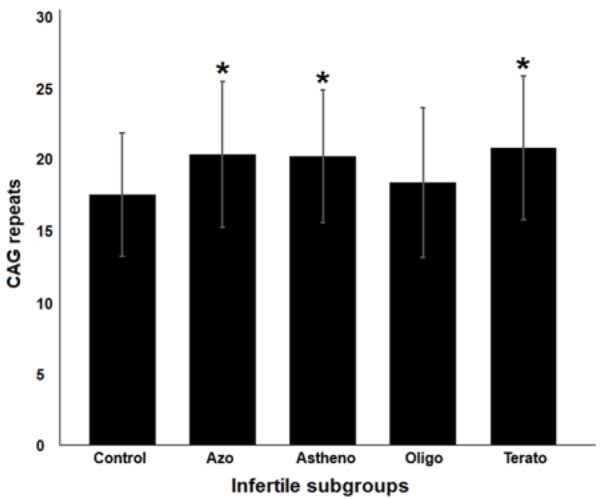
The association results of CAG repeats with infertile subtypes; significant results are shown with star sign.

To estimate androgen receptor CAG repeats length distribution in cases and controls, the mean (M), standard deviation (SD), and *p*-value (*p*) were calculated, results of which are summarized in Table 2(Tab. 2). Our findings were indicative of a significant association between increased length of repeats and the risk of male infertility (*p*< 0.0001; Figure 2(Fig. 2)). Moreover, the case-control study was stratified into four subgroups of azoospermia, asthenozoospermia, oligozoospermia, and teratozoospermia based on sperm parameters. As depicted in Figure 3(Fig. 3), significant associations were found between the increased number of CAG repeats and the risk of male infertility in azoospermia (*p*= 0.048), asthenozoospermia (*p*= 0.013), and teratozoospermia (*p*= 0.002) subgroups.

### Meta-analysis

**Figure 4:**
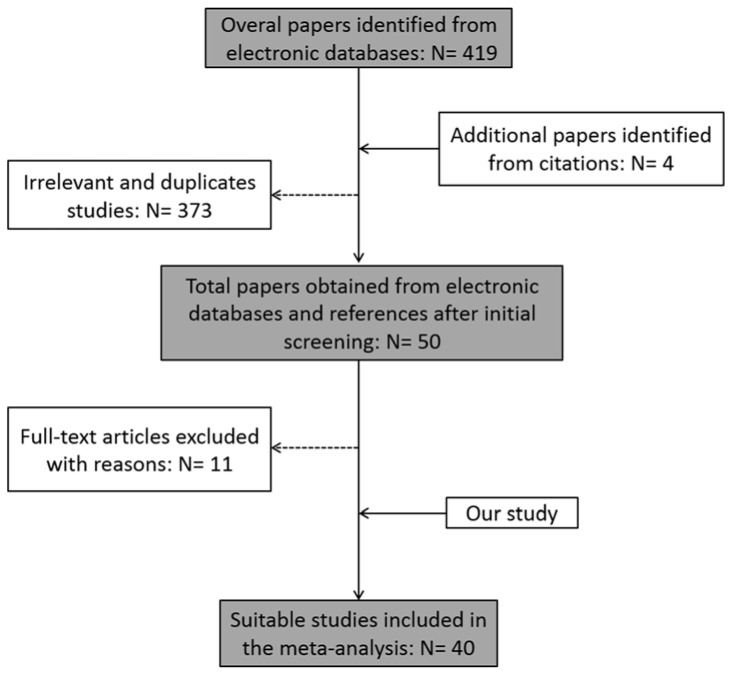
Study selection flowchart

Figure 4(Fig. 4) presents the flowchart for paper selection. By the initial search, 419 papers were collected and 4 new articles were found from the references of collected studies. From these articles, 373 were excluded due to being irrelevant or duplicate. Following the screening process, 11 studies were removed due to reporting insufficient data or being a meta-analysis or review article. Finally, 39 studies were identified as eligible studies and were added to the meta-analysis along with our data. Among these studies, 20 projects belonged to Caucasians, 9 studies were performed in Asians, and 4 others were carried out in the African population.

**Figure 5:**
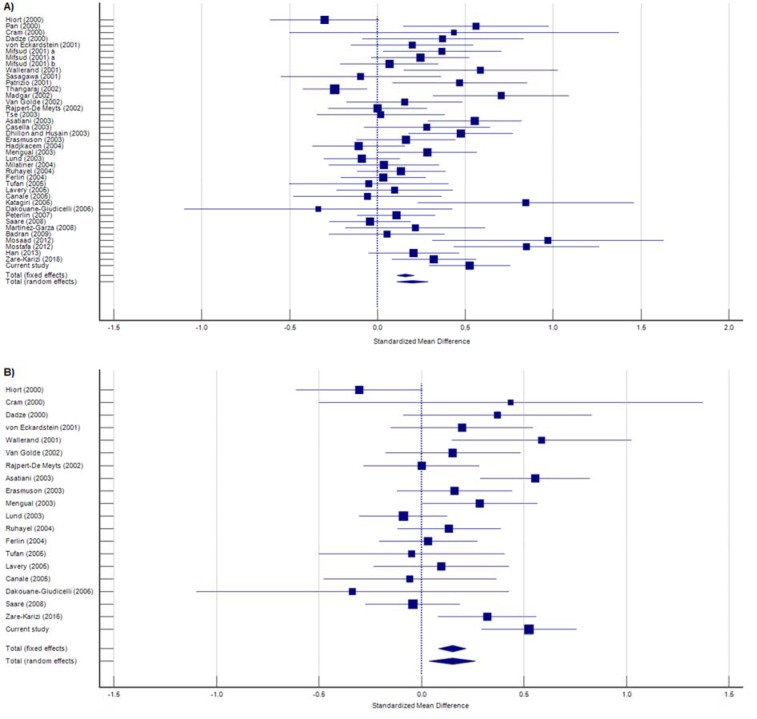
Forest plot for the association of *AR*-CAG polymorphism with male infertility in overall (A) and Caucasian (B) analyses

**Figure 6:**
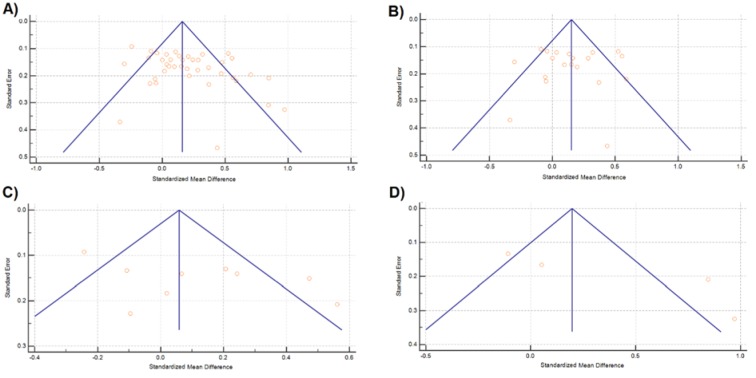
Funnel plot for the association of *AR*-CAG polymorphism with male infertility in overall (A), Caucasian (B), Asian (C), and African (D) analyses

Based on Table 3(Tab. 3), the primary meta-analysis focused on the overall analysis. Analysis of the total set of 40 studies demonstrated statistically significant longer CAG repeat length in all infertile men, compared to the control group (SMD= 0.199, 95 % CI= 0.112-0.287, *p*< 0.001; Figure 5A(Fig. 5)). In addition, the subgroup analysis was stratified by ethnicity divided into Asian, Caucasian, and African ethnicities. In the Caucasian population, a significant association was found between increased length of CAG repeats and the probability of male infertility (SMD= 0.151, 95 % CI= 0.040-0.263, *p=* 0.008; Figure 5B(Fig. 5)). Nevertheless, no such correlation was found in the Asian (SMD= 0.113; 95 % CI= -0.071-0.296, *p*= 0.228) and African (SMD= 0.394, 95 % CI= -0.109-0.896, *p*= 0.124) populations. A true heterogeneity was observed in overall analysis (*I*^2^= 66.97 %, *P*_heterogeneity_< 0.0001), which remained after stratification analysis in the Caucasian (*I*^2^= 61.09 %, *P*_heterogeneity_= 0.0002), Asian (*I*^2^= 72.44 %, *P*_heterogeneity_= 0.0003), and African (*I*^2^= 85.90 %, *P*_heterogeneity_= 0.0001) subgroups. Moreover, there was no significant publication bias in overall (*P*_Egger_= 0.279) and stratified (Caucasian: *P*_Egger_= 0.844; Asian: *P*_Egger_= 0.227; African: *P*_Egger_= 0.682) meta-analysis (Figure 6(Fig. 6), References in Figure 6: Asatiani et al., 2003[3]; Badran et al., 2009[4]; Canale et al., 2005[7]; Casella et al., 2003[9]; Cram et al., 2000[11]; Dadze et al., 2000[12]; Dakouane-Giudicelli et al., 2006[13]; Dhillon and Husain, 2003[15]; Erasmuson et al., 2003[17]; Ferlin et al., 2004[18]; Hadjkacem et al., 2004[22]; Han et al., 2013[23]; Hiort et al., 2000[24]; Katagiri et al., 2006[27]; Lavery et al., 2005[30]; Lund et al., 2003[32]; Madgar et al., 2002[33]; Martínez-Garza et al., 2008[34]; Mengual et al., 2003[35]; Mifsud et al., 2001[37]; Mifsud et al., 2001[36]; Milatiner et al., 2004[38]; Mosaad et al., 2012[39]; Mostafa et al., 2012[40]; Pan et al., 2002[47]; Patrizio et al., 2001[49]; Peterlin et al., 2007[50]; Rajpert-De Meyts et al., 2002[52]; Ruhayel et al., 2004[53]; Saare et al., 2008[54]; Sasagawa et al., 2001[55]; Thangaraj et al., 2002[58]; Tse et al., 2003[59]; Tufan et al., 2005[60]; Van Golde et al., 2002[61]; von Eckardstein et al., 2001[62]; Wallerand et al., 2001[63]; Zare-Karizi et al., 2016[66]).

## Discussion

Male infertility is an important problem and one of the causes of couples' inability to bear children (Ge et al., 2014[19]). It is estimated that there are about 30 million infertile men in the world (Agarwal et al., 2015[1]) and 15-30 % of causes of male infertility refer to genetic factors (Talebi et al., 2018[57]). Therefore, identifying the genetic risk factors of idiopathic male infertility will be very important. Genetic polymorphisms in genes affecting the process of spermatogenesis could be considered as a possible risk factor for male infertility (Rafatmanesh et al., 2018[51]). In the current research, we evaluated the possible role of androgen receptor-CAG trinucleotide repeat length in idiopathic male infertility through a case-control followed by a meta-analysis. Our findings revealed that the risk of male infertility increased with elevating the length of androgen receptor-CAG trinucleotide repeat. However, previous studies reported conflicting results (Delli Muti et al., 2014[14]; Nenonen et al., 2010[42]; Khan et al., 2018[28]). Therefore, we employed a meta-analysis approach to achieve more comprehensive and conclusive results. The meta-analysis showed a significant association between androgen receptor-CAG length and male infertility. In addition, stratified meta-analysis demonstrated that the mentioned polymorphism could be a risk factor for male infertility just in the Caucasian population. This could explain the interactive effect of ethnicity and geographical factors on *AR*-CAG variation. The meta-analysis confirmed a true heterogeneity among studies, which was remaining after stratification. Therefore, ethnicity could not be the source of heterogeneity and it should be explored by other subgroups such as sample size, phenotypes of infertility, and the source of controls. Possible publication bias was judged by funnel plot and Egger test, presenting no bias in our meta-analysis. Therefore, the results of the pooled data could be considered reliable.

Androgens play an essential role in puberty and male fertility. In addition, they are required for the growth of male reproductive organs, such as epididymis, seminal vesicle, vas deferens, prostate, and the penis. Androgens carry out their effects through androgen receptor that are essential for spermatogenesis (Nilsson et al., 2015[43]). Intratesticular testosterone secreted by Leydig cells is mainly bound to the androgen receptor, and stimulation of the receptor will lead to the initiation and maintenance of the spermatogenic process and inhibition of germ cell apoptosis. Severe deficiencies of the AR may result in abnormal male sexual growth (Dohle et al., 2003[16]). Our study is biologically reasonable due to showing the association of *AR*-CAG variation with the risk of male infertility. One of the hazardous regions on exon 1 of the human *AR* gene coding stretches of identical glutamine residue with polymorphic length variation (Grigorova et al., 2017[21]). Changes in the structure of the androgen receptor with 40 or more glutamine residues, as in Kennedy's syndrome, result in the aggregation of insoluble protein (Stenoien et al., 1999[56]). However, the effects of polyQ length variations within the normal range on the function of AR and impact on the development of the non-neurological disease have not been exactly explained. While CAG repeat polymorphism within the normal range could not affect the affinity of androgen with AR (Chamberlain et al., 1994[10]), there is an inverse association between the length of polyQ and AR transactivation capacity (Irvine et al., 2000[25]; Buchanan et al., 2004[6]). In addition, the role of AR-CAG repeat in some diseases was assessed in a study, proposing three mechanisms of pathogenesis, as follows: 1- loss of function of protein, 2- gain of function of the protein, and 3- gain of function of RNA containing CUG repeats (La Spada and Taylor, 2010[29]). It is suggested that the role of these three mechanisms in male infertility must be further explored in future studies. Some recent reports have investigated the effects of genetic polymorphisms on molecular aspects of protein and RNA by in silico tools (Karimian et al., 2018[26]; Nejati et al., 2018[41]; Zamani-Badi et al., 2018[65]; Noureddini et al., 2018[44]). 

One of the major drawbacks of the present study was lack of evaluation of the gene-gene and gene-environmental interactions. Another limitation was restricting the search procedure in English in the meta-analysis, which might have led to language bias. Furthermore, there was no access to original data (e.g., age, BMI, smoking status, and drinking status) from the included studies to adjust our data to them.

## Notes

Mohammad Karimian and Hossein Nikzad (hnikzad10@gmail) contributed equally as corresponding authors.

## Conflict of interest

The authors declare that there is no conflict of interest regarding the publication of this paper.

## Funding

This study was supported by Grants from the Kashan University of Medical Sciences (No. 96023).

## Figures and Tables

**Table 1:**
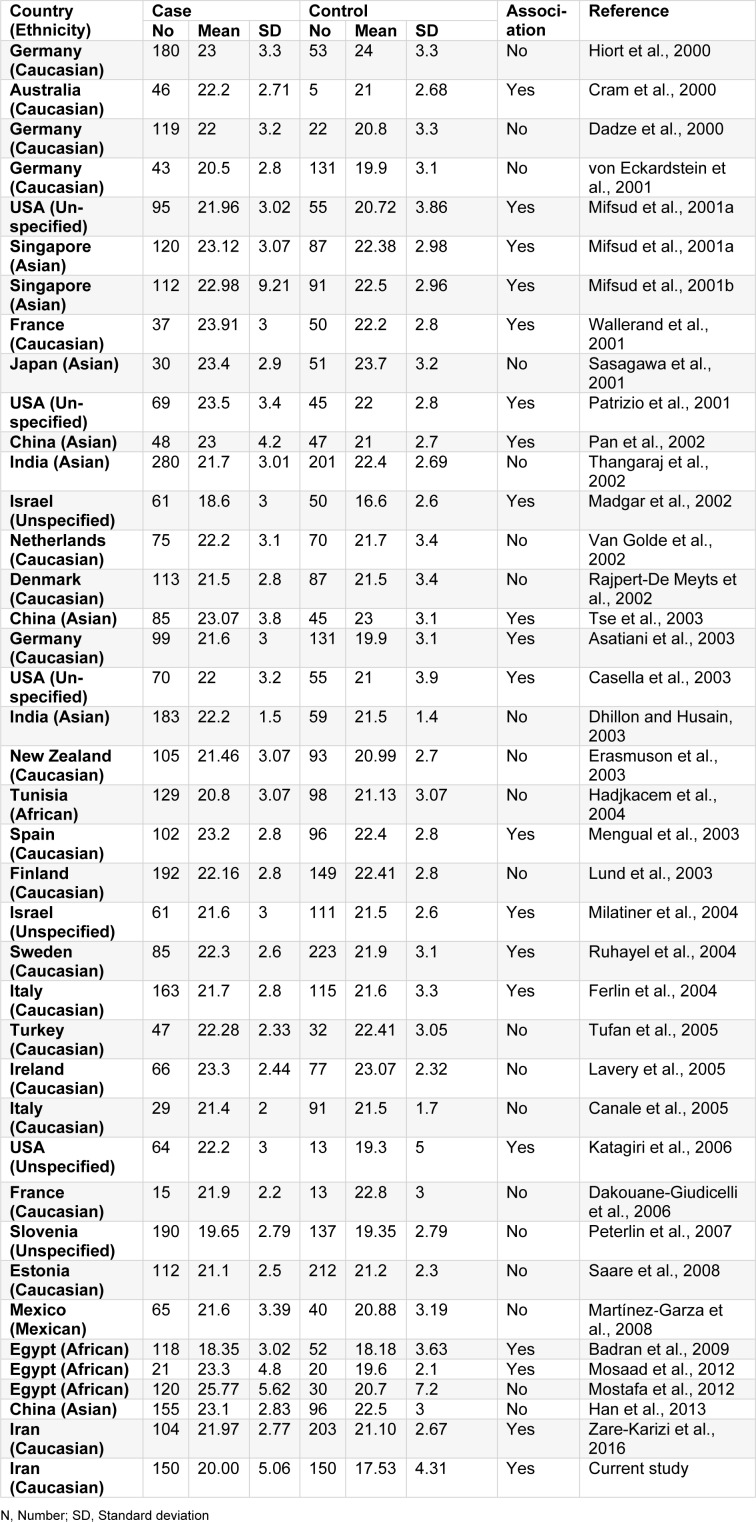
Characteristics of the case-control studies included in the meta-analyses

**Table 2:**
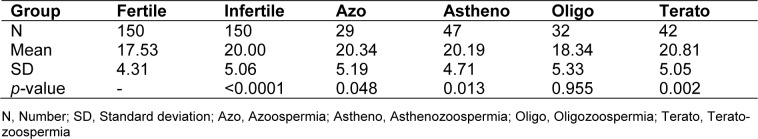
Results of overall and stratified case-control study

**Table 3:**
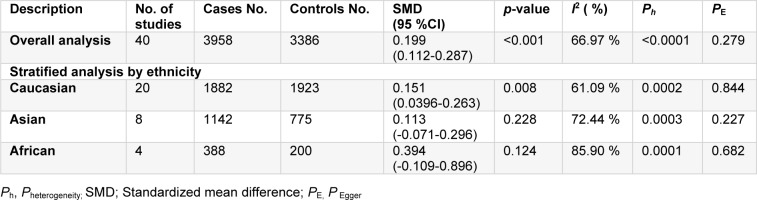
Results of overall and stratified meta-analyses
